# Oropharyngeal Colostrum Positively Modulates the Inflammatory Response in Preterm Neonates

**DOI:** 10.3390/nu12020413

**Published:** 2020-02-05

**Authors:** Estefanía Martín-Álvarez, Javier Diaz-Castro, Manuela Peña-Caballero, Laura Serrano-López, Jorge Moreno-Fernández, Belen Sánchez-Martínez, Francisca Martín-Peregrina, Mercedes Alonso-Moya, José Maldonado-Lozano, Jose A. Hurtado-Suazo, Julio J. Ochoa

**Affiliations:** 1 Unit of Neonatology, Pediatric Service, Hospital Universitario Materno-Infantil Virgen de las Nieves, 18014 Granada, Spain; estenia.martin.alvarez@gmail.com (E.M.-Á.); mapeca06@yahoo.es (M.P.-C.); lserranolopez@hotmail.com (L.S.-L.); belensamar@gmail.com (B.S.-M.); paquimarpe@gmail.com (F.M.-P.); malonsomo@gmail.com (M.A.-M.); jahsuazo@yahoo.es (J.A.H.-S.); 2 Department of Physiology, University of Granada, 18071 Granada, Spain; jorgemf@ugr.es (J.M.-F.); jjoh@ugr.es (J.J.O.); 3 Institute of Nutrition and Food Technology “José Mataix Verdú”, University of Granada, 18071 Granada, Spain; 4 Pediatrics Department, Virgen de las Nieves University Hospital, University of Granada, 18071 Granada, Spain; jmaldon@ugr.es; 5 Institute of Biosanitary Research of Granada, Maternal and Child Health Network, Carlos III Institute, 28020 Madrid, Spain

**Keywords:** colostrum administration, premature neonates, inflammation, clinical outcomes

## Abstract

During the first days of life, premature infants have physiological difficulties swallowing, thereby missing out on the benefits of breastfeeding. The aim of this study is to assess the effects of oropharyngeal mother’s milk administration in the inflammatory signaling of extremely premature infants. Neonates (*n* = 100) (<32 week’s gestation and/or <1500 g) were divided into two groups: mother’s milk group (*n* = 48), receiving 0.2 mL of oropharyngeal mother’s milk every 4 h for the first 15 days of life, and a control group (*n* = 52), not receiving oropharyngeal mother’s milk. Serum concentrations of interleukin (IL) IL-6, IL-8, IL-10, IL-1ra, tumor necrosis factor alpha (TNF-α), and interferón gamma (IFN-γ) were assessed at 1, 3, 15, and 30 days of postnatal life. Maternal and neonatal outcomes were collected. The rate of common neonatal morbidities in both groups was similar. The mother’s milk group achieved full enteral feeding earlier, and showed a decrease in Il-6 on days 15 and 30, in IL-8 on day 30, and in TNF-α and INF-γ on day 15, as well as an increase in IL-1ra on days 3 and 15 and in IL-10 on day 30. Oropharyngeal mother’s milk administration for 15 days decreases the pro-inflammatory state of preterm neonates and provides full enteral nutrition earlier, which could have a positive influence on the development of the immune system and inflammatory response, thereby positively influencing other developmental outcomes.

## 1. Introduction

Inflammation is implicated in a high proportion of preterm births [1], and is associated with fetal inflammatory response syndrome (FIRS). This syndrome is characterized by systemic inflammation and is associated with the development of long-term sequelae by multifunctional organic failures such as sepsis neonatal, bronchopulmonary dysplasia, intraventricular hemorrhage, and necrotizing enterocolitis (NEC), thereby increasing perinatal and neonatal mortality and morbidity [2]. During pregnancy, especially in preterm neonates, several developing tissues are especially vulnerable and are profoundly affected by cytokines that circulate rapidly through fetal blood, potentially causing inflammatory signaling, which can trigger intracellular signaling cascades that result in organ damage and neonatal morbidity [3]. Under these conditions, the most vulnerable tissues are lung, brain, and intestine [4,5]. Taking into account the importance of inflammation regarding morbidity and/or neonatal mortality, it is important to obtain all possible information about inflammatory signaling in newborns [6,7,8,9].

Mother’s milk is the best first immune stimulator in infants, featuring the perfect species-specific nutrition, because it contains many types of protective agents and enhances neurodevelopmental outcomes [10,11]. These effects are attributed to a multitude of protective (immune and trophic) biofactors [11,12,13]. Milk biofactors protect against infection, providing antimicrobial, anti-inflammatory, and immunomodulatory functions, preventing adherence to the gastrointestinal mucosa of pathogens, improving gastrointestinal microbiota, keeping the integrity of the intestinal barrier and repairing injured areas, promoting intestinal motility and maturation, and providing antioxidant defense [10,12,13].

Despite the importance of breastfeeding in the development of the newborn’s immune system, in some cases, this feeding is not possible in the first days of life, especially in premature infants, due, among others causes, to the existence of physiological difficulties in swallowing. Therefore, it is of great interest to search for noninvasive mechanisms that allow these neonates to receive the advantages of breast milk; the administration of oropharyngeal mother’s milk is a safe and well tolerated intervention with many clinical benefits. In this clinical intervention, with even extremely preterm babies [14,15], small volumes of mother’s milk, especially colostrum, are directly dropped onto the oropharyngeal mucosa [16]. Although many studies support the clinical implications of this safe clinical intervention, there is scarce evidence of its effects on inflammatory signaling and/or its clinical benefits. It is therefore necessary to increase the study population in order to draw more reliable conclusions [15,17,18]. In addition, to date, published studies have supplied colostrum only during the first days of life and in preterm infants of less than 28 weeks’ gestation. For to these reasons, it is necessary to deepen our understanding and clarify the clinical implications related to this practice; therefore, the aim of this study is to assess the effects of oropharyngeal administration of mother’s milk. This is the first study to characterize the serum pro- and anti-inflammatory biomarker profiles of extremely premature infants.

## 2. Methods

### 2.1. Experimental Design and Subjects

**Figure 1:**
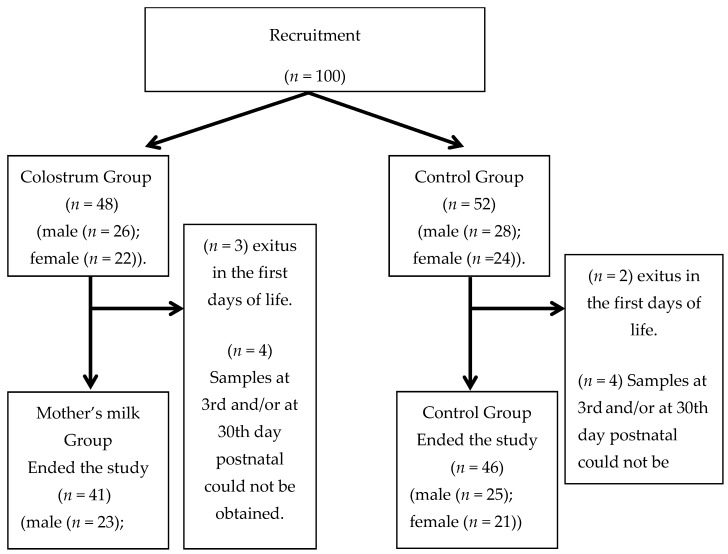
Flowchart showing participant progress and dropouts in the study.

Informed consent was obtained from the parents before they participated in the study. The study was conducted in accordance with the Declaration of Helsinki, and the protocol was approved by the Ethics Committee of the University Hospital “Virgen de las Nieves” (PI-0374-2014). Inclusion criteria were extremely preterm infants in the Neonatal Intensive Care Unit with <32 weeks’ gestation and/or with a weight below 1500 g at birth. Exclusion criteria were chromosomopathies or congenital abnormalities, consumption or intake more than 10 mcg/kg/min of vasoconstrictive drugs, and/or HIV-positive mother. Neonates (*n* = 100) were divided into two groups: the mother’s milk group (*n* = 48) receiving mother’s milk via oropharynx, and the control group (*n* = 52), which did not receive oropharyngeal mother’s milk, because it was not available in the first 24 h of life. Figure 1 shows the flowchart of the infants involved in this study and the reasons for the dropouts.

### 2.2. Milk Sampling and Administration

The protocol for obtaining and administering breast milk has been previously described [19]. After being informed by the researchers in the first 24 h postpartum, the mothers had to obtain mother’s milk every 2 to 3 h by electric pumping (Mendela, Baar, Switzerland) (at least eight times every 24 h). Mother’s milk was collected in prelabeled, sterile vials, and then a trained nurse administered the milk to the neonates through sterile syringes (BD, Franklin Lakes, NJ, USA) via the oropharyngeal route. Each day, syringes were prepared with 0.2 mL mother’s milk, labelled, and stored at 4 °C in labelled plastic cups. Prior to the administration of the milk, the syringe was heated for 5 min in the infant’s incubator. For at least 2 min, the nurse administered 100 µL of the mother’s milk on one side of the oral mucosa, and then another 100 µL on the other side in order to maximize oropharynx absorption (a total of 200 µL). The intervention was repeated every 4 h for the next 15 days. During the intervention, neonatal welfare was monitored, controlling any sign of discomfort (tachycardia, bradycardia, tachypnoea, bradypnea, PO_2_, and changes in blood pressure), and in case of alterations, the procedure stopped. The enteral nutrition of each patient, regardless of the assigned group, was decided by the medical team responsible for their care. The trophic enteral nutrition began in the first 24–48 h, i.e., as soon as possible, if there were no contraindications.

### 2.3. Sample Collection and Analysis

Four blood samples were obtained during the first 30 days of postnatal life to evaluate the influence on the inflammatory signaling: at enrolment (M1), on the 3rd day (M2), on the 15th day (M3) and on the 30th day (M4). The serum was stored after being aliquoted at −80 °C until further use.

### 2.4. Maternal and Neonatal Outcomes

Mother variables comprised type of birth, prenatal corticosteroids, prenatal antibiotics, prenatal amniotic infection risk, preterm outcomes (sex, Apgar score, weight, height and head circumference at birth, weight gaining first month and Clinical Risk Index for Babies (CRIB)), time to achieve enteral feeding (at least 100–120 mL/kg/day), volume of enteral feeding, volume of parenteral feeding, and modifications in cerebral ultrasound (classification by de Vries et al. [20]), NEC grade greater or equal to II according to the modified Bell’ staging classification [21], or proven sepsis [22].

### 2.5. Inflammatory Parameters Measurement

The pro- and anti-inflammatory parameters studied (IL-6, IL-8, IL-10, IL-1ra, TNF-α, and IFN-γ) were determined using a Multiplex panel (Human Sepsis Magnetic Bead Panel 3, HTH17MAG- 14K) based on the luminex xMAP technology (Merck Millipore, Boston, MA, USA). Cytokine concentrations in plasma samples were determined by comparing the mean of duplicate samples with the standard curve for each assay.

### 2.6. Statistical Analysis

The results are presented as the mean ± standard error of mean (SEM). The sample size calculation has been previously described [19]. The Kolmogorov-Smirnoff’s and Levene’s tests were used to check the normality and homogeneity of variance, respectively. To compare the baseline characteristics between the case and control groups, the Student *t* test for independent samples or Mann-Whitney test were used for the numerical variables in case of nonnormality. Categorical variables were checked using the chi-square Pearson or Fisher test when the conditions of applicability were not met. To assess the effect of the oropharyngeal administration and the time evolution for each variable studied in each group, a general linear model for repeated measures procedure was applied, with Bonferroni correction for multiple comparisons (the *p*-values were corrected considering six possible comparisons). A Bonferrini’s test allowed us to determine the intra- and inter- subject differences. A value of *p* < 0.05 was considered significant. The SPSS version 21.0 (SPSS Statistics for Windows, SPSS INC., Chicago, IL, USA) software was used for data analysis.

## 3. Results

### 3.1. Maternal and Neonatal General Characteristics

Neonatal and maternal general characteristics are shown in Table 1. No differences were recorded in the different period of the study for the mothers or neonates, including volumes of enteral and parenteral feeding supplied in the different periods of study. Regarding sex differences, there were no significant differences between groups. In relation to smoking, we have no record that mothers smoked during pregnancy, according to the records in the anamnesis of the mothers.

### 3.2. Clinical Outcomes

The clinical outcomes of preterm infants are featured in Table 2. Babies receiving mother’s milk achieved full enteral feeding sooner than the control group (*p* < 0.05).

### 3.3. Pro- and Anti-Inflammatory Interleukins

**Figure 2:**
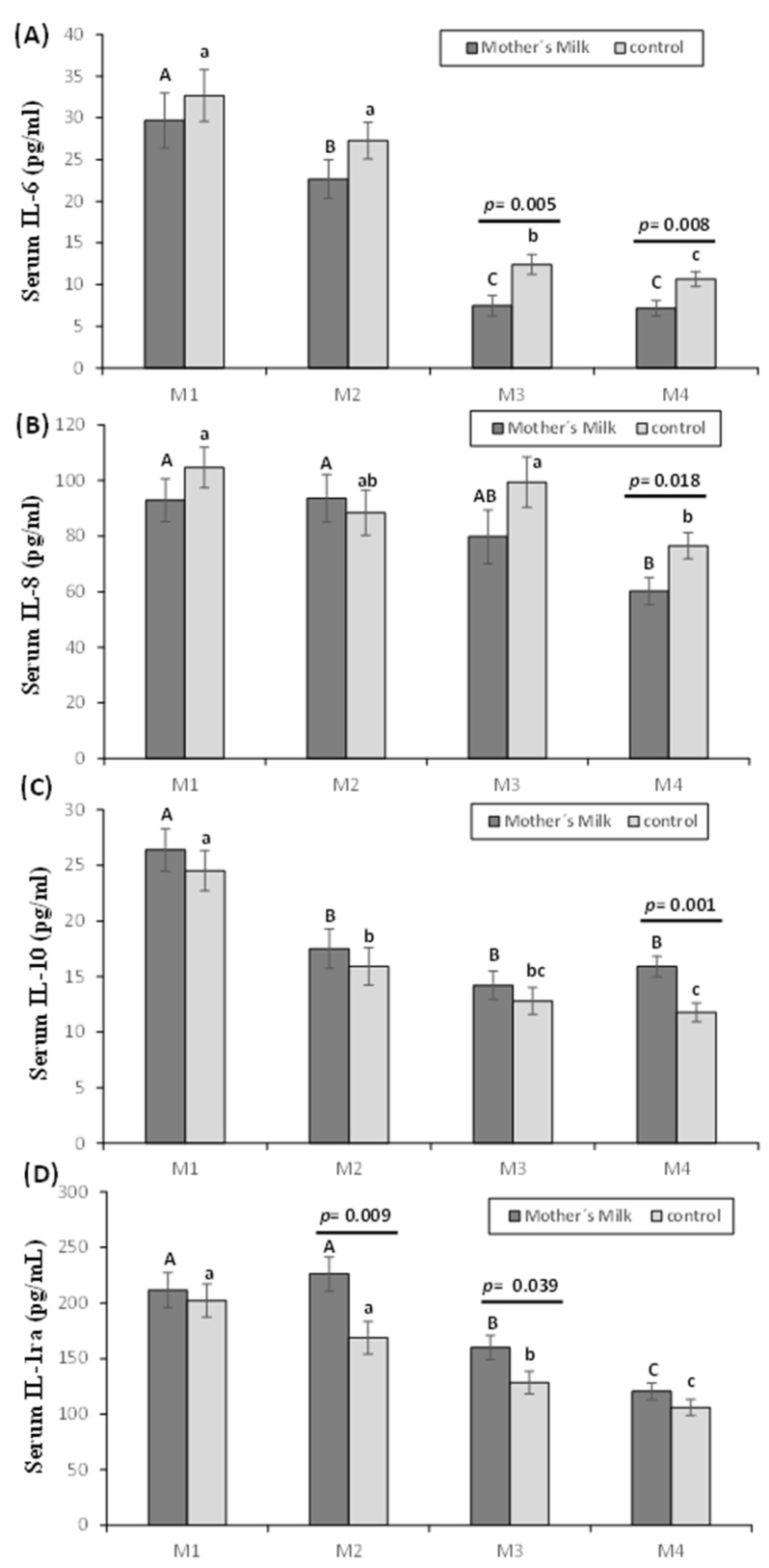
Effect of oropharyngeal mother’s milk administration on the levels of IL-6 (**A**), IL-8 (**B**), IL-10 (**C**), and IL1-ra (**D**) in serum. Results are expressed as mean ± standard error of the mean. A line between bars means statistically significant differences between groups (*p* < 0.05). Different letters in every group indicate significant differences due to the time (mother’s milk (A, B, C), control (a, b, c) (*p* < 0.05)). M1: Birth (basal value), M2: 3rd day of postnatal life; M3: 15th day of postnatal life, M4: 30th day of postnatal life.

Serum pro- and anti-inflammatory interleukins are summarized in Figure 2. IL-6 was lower after 15 and 30 days of postnatal life in the mother’s milk group compared to the control group (*p* < 0.05) (Figure 2A). With regard to the evolution in both groups, a decrease was observed in M3 and M4, compared to M1 and M2 (*p* < 0.05), and in the mother’s milk group, a decrease in M2 with regard to M1 was also observed (*p* < 0.05). IL-8 was lower in the first month of life in the oropharyngeal mother’s milk compared to the control group (*p* < 0.05). In addition, IL-8 decreased in the mother’s milk group after 30 days postnatal life compared to M1 and M2 (*p* <0.05); in the control group, this decrease was also observed in M4, but compared with M1 and M3 (*p* < 0.05) (Figure 2B).

The mother’s milk group showed higher IL-10 levels, being statistically significant after 1 month (*p* < 0.05). IL-10 was lower in M2, M3, and M4 compared to M1 in the mother’s milk group (*p* < 0.05). In the control group, a significant decrease was observed in M4 compared to M1 and M2 (*p* < 0.05), and in M3 compared to M1 (*p* < 0.05) (Figure 2C). IL-1ra levels (Figure 2D) were lower in the control group compared to the mother’s milk group, being statistically significant in M2 and M3 (*p* < 0.05). IL-1ra in both groups followed the same trend, decreasing in M4 compared to M1, M2, and M3 (*p* < 0.05), and in M3 compared to M1 and M2 (*p* < 0.05).

### 3.4. Other Cytokines: TNF-α and INF-γ

**Figure 3:**
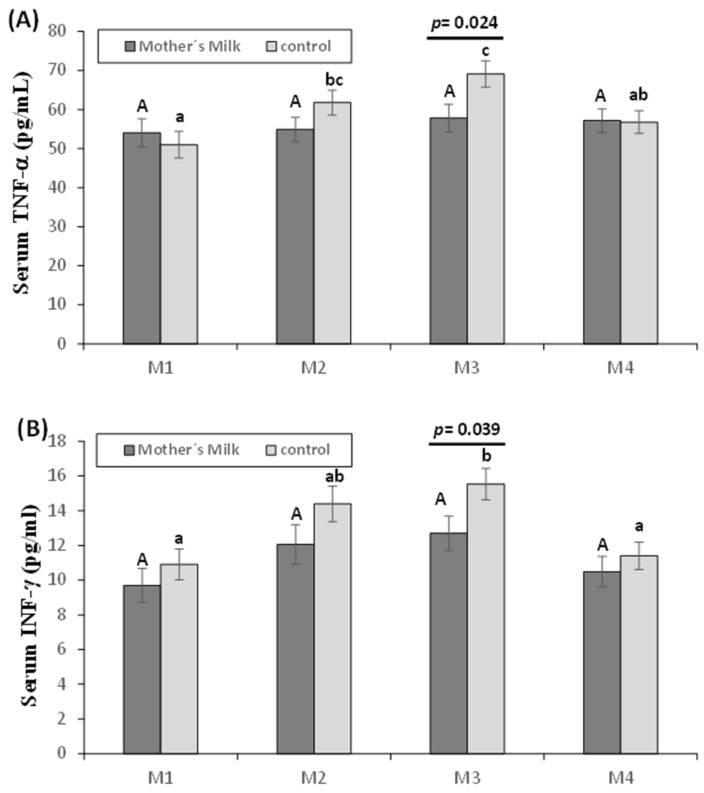
Effect of oropharyngeal mother’s milk administration on the levels of TNF-α (**A**) and INF-γ (**B**) in serum. Results are expressed as mean ± standard error of the mean. A line between bars means statistically significant differences between groups (*p* < 0.05). Different letters in every group indicate significant differences due to the time (mother’s milk (A, B, C), control (a, b, c) (*p* < 0.05)). M1: Birth (basal value), M2: 3rd day of postnatal life, M3: 15th day of postnatal life, M4: 30th day of postnatal life.

Figure 3 shows the values of TNF-α (Figure 3A) and INF-γ (Figure 3B). These cytokines were higher in the control group at 15 days postpartum compared to those in the mother’s milk group (*p* < 0.05). Both cytokines showed no differences in their evolution in the mother’s milk group; however, in the control group, an increase in M3 was observed when comparing to M1 and M4 in both cytokines (*p* < 0.05), and in M2 compared to M1 in TNF-α (*p* < 0.05).

## 4. Discussion

Breast milk, especially mother’s milk, is the perfect species-specific nutrition for preterm infants, because it has different types of bioprotective agents; however, in some cases, breastfeeding is limited, and the administration of oropharyngeal mother’s milk has been shown to be a safe means of providing part of its benefits to premature newborns [19,23]. However, there are still limitations and controversies in the scientific literature, because the results are often inconclusive due to small sample sizes [14], the use of animal models [24], and the use of secretions (urine and saliva) instead of blood [14,15,17] to perform the assessments. Taking into account these limitations, together with the lack of information about inflammatory signaling, the current study was designed to include a high number of premature neonates, being the first such study to characterize the pro- and anti-inflammatory biomarker profiles of premature neonates in serum, as opposed to in other secretions such as urine and saliva after the administration of mother’s milk, which strengths the results presented herein.

One of the controversies observed in the literature is focused on the time to achieve complete enteral nutrition after the administration of oropharyngeal mother’s milk; in this sense, the study conducted by Rodriguez et al. [25] reports differences between groups; in contrast, Zhang et al. [15] did not observe such differences. However, in all cases, the studies were performed with a smaller population than the current study. In the current study, preterm infants receiving mother’s milk achieved full enteral nutrition earlier than the control group. These results are noteworthy, because the inability to obtain enteral nutrition in the first days of life leads to villous atrophy and delayed nutrient absorption, increasing the risk of inflammation, NEC, and hospital-related infections [14]. In this sense, the oropharyngeal administration of mother’s milk could be considered a potential “immune therapy” for preterm neonates during the first days of life in which they have little tolerance for enteral feeding.

In relation to inflammatory signaling, the main focus of this study, we observed that preterm neonates who received oropharyngeal mother’s milk showed lower levels of pro-inflammatory cytokines (IL-6, IL-8) and higher expression of anti-inflammatory cytokines (IL-10 and IL-1ra). The only study on this topic to date with a similar number of cytokines did not show many differences between the two study groups [17], although the measurements were performed on urine and saliva, with a smaller population and a shorter administration period.

Inflammation is involved in a significant number of preterm births, despite of the presence or absence of infection [1]; it is related to the development of fetal inflammatory response syndrome, which is associated with the development to long-term sequelae due to multifunctional organ compromise such as neonatal sepsis, bronchopulmonary dysplasia, intraventricular hemorrhage, NEC, etc. [2]. Therefore, systemic inflammation in newborns could be considered an independent risk factor of morbidity, affecting several organs such as lung, intestine, and brain [2,26].

One of the most studied pro-inflammatory cytokines is IL-6, which has pleiotropic effects involving the activation of the acute phase of the immune response; it has also been used as a hallmark of the fetal inflammatory response syndrome and early neonatal sepsis [27]. This cytokine decreased progressively in all the neonates in the current study, reflecting the development of the immune system and the adaptive response of the neonate to the extrauterine conditions. However, the preterm neonates receiving oropharyngeal milk for one month showed a higher decrease of this cytokine from the 15th day of life, which could represent an advantage for preterm neonates, because inflammation is a common upstream pathway observed in major perinatal diseases [2,26].

Another early marker of neonatal sepsis is IL-8, a proinflammatory and chemotactic cytokine, which increases its expression in situations of homeostatic alterations caused by infection, trauma, and other conditions [28,29]. An important component in the inflammatory response and innate immune system is the capacity of IL-8 to attract neutrophils; therefore, the decrease recorded in preterm neonates after one month of mother’s milk administration indicates a positive influence on the neonate’s developing immune system and inflammatory response. Lee et al. [17] also observed a decrease in the levels of IL-8, although this was recorded in saliva and in the first 15 days of life. One of the possible causes of this lower expression is a decrease in the stimulation of this cytokine due to the increase in the Il-1ra observed in the current study, which blocks the stimulation of IL-8 by IL-1β [30].

The cytokine IL-10, as an immune-regulator, suppresses antigen-specific T-cell proliferation and inhibits Th1 responses, increasing the survival and proliferation of B cells. Although its concentration in mother’s milk is not too high, its level does not decline in human milk over time [31], helping preterm neonates to cope with the inflammation that is triggered by several stressors. In addition, mother’s milk from mothers delivering preterm babies is more caloric and contains higher levels of protein, fat, and bioactive factors including IL-10 [32]. Reports about this cytokine show some controversy, and in this sense, high concentrations of IL-10 in serum are related to greater severity of pathologies such as respiratory distress [33]; however, low levels of IL-10 have been associated with an increased risk of developing bronchopulmonary dysplasia [33]. In addition, it has been shown to be an important inflammatory mediator in neonatal sepsis, since it plays an important role in the prevention of excess in the inflammatory response during this life-threatening clinical condition. In our study, we observed a higher IL-10 concentration in the mother’s milk group at 30 days of age, which, together with the decrease in IL-6, could have a beneficial effect, since it has been observed that a high IL-6/IL-10 ratio is found in patients with worse prognoses [29]. Similar reports to the current study did not report any effect of the administration of mother’s milk on this cytokine, although the population and time of mother’s milk administration were smaller [17].

Another pro-inflammatory cytokine is TNF-α, which acts as one of the main mediators of septic shock in neonates, as well as other tissue damage. Its production is defective in term neonates [29], and its receptors in their soluble forms, TNF-RI and TNF-RII, are present in human milk [34]. In the current study, we observed an increase of TNF-α in the control group until day 15 of life, which could reflect the inflammatory response syndrome in the premature newborn. However, this increase was not observed in the mother’s milk group, which showed a lower concentration at 15 days of age. According to Castellote et al. [35], TNF-RI levels in mother’s milk can act by regulating the biological effect of TNF-α; therefore, it is responsible for some of the anti-inflammatory effects of mother’s milk.

Finally, IFN-γ in the control group showed a similar trend to that shown by TNF-α with an increase until day 15 that was not observed in the mother’s milk group. From the point of view of the preterm survival rate, this IFN-γ downstream would be clearly beneficial, because in a study of ventilated preterm infants, the IFN-γ level was higher within the first 48 h of life in infants that developed bronchopulmonary dysplasia or died [36,37].

Additionally, together with the beneficial effects on the inflammatory signaling that could be obtained by the administration of oropharyngeal mother’s milk, preterm neonates receiving this clinical intervention achieved complete enteral nutrition sooner. The lower time to reach full enteral nutrition influences populations of beneficial microorganisms in the infant gut [38], improving the colonization of bacteria and the development of the preterm digestive system, resulting in a decreased release of cytokines, which may positively influence clinical outcomes.

As a secondary objective, in this study, we assessed the effect of oropharyngeal mother’s milk on the incidence of NEC, proven sepsis, and other clinical outcomes. Our results show an absence of difference between the groups. We consider that this absence is due to the low prevalence of these pathologies, that would require a higher number of premature infants.

## 5. Conclusions

The current study proposes that the administration of oropharyngeal mother’s milk in the first month of life contributes to decreasing the pro-inflammatory state of the preterm neonate, indicating a beneficial influence on the inflammatory response. Moreover, preterm infants receiving mother’s milk via oropharynx achieved complete enteral nutrition sooner than babies who did not, representing a metabolic advantage for the underdeveloped gastrointestinal system; this could minimize comorbidities linked to nutrient absorption in this population. These findings have implications for the development of the preterm neonate, wherein inflammation plays a pathophysiological role, associated with adverse neonatal outcomes independently of the duration of gestation.

## Figures and Tables

**Table 1:** Maternal and neonatal general characteristics.

Maternal and Neonatal Characteristics	Units	Mother’s Milk Group	Control Group	*p*-Value
Type of birth	Vaginal (%)	47.5	34.8	0.33
	Cesarean (%)	52.5	65.2	
Prenatal corticosteroids	No (%)	12.5	10.9	0.47
	Yes (%)	89.1	87.5	
Prenatal antibiotics	No (%)	20.5	15.2	0.49
	Yes (%)	76.9	84.8	
Prenatal amniotic infection risk *	No (%)	32.5	39.1	0.41
	Yes (%)	67.5	60.9%	
Gestational age (weeks)		29.9 ± 0.4	29.5 ± 0.3	0.34
Sex	Male (%)	60.0	56.5%	0.91
	Female (%)	40.0	43.5%	
Apgar 1	min	6.4 ± 0.3	6.6 ± 0.3	0.65
Apgar 5	min	8.1 ± 0.2	8.1 ± 0.2	0.83
Weight	g	1230.1 ± 48.2	1267.6 ± 52.4	0.60
Height	cm	38.6 ± 0.5	38.4 ± 0.7	0.69
Head circumference	cm	27.1 ± 0.4	27.0 ± 0.4	0.83
Weight gaining first month	g	612.9 ± 31.7	527.4 ± 49.2	0.06
CRIB Score		2.2 ± 0.4	2.0 ± 0.4	0.17
Enteral Feeding	mL/kg/day			
M2		39.1 ± 3.8	34.5 ± 4.4	0.53
M3		152.1 ± 9.9	162.2 ± 6.3	0.21
M4		160.8 ± 5.7	166.8 ± 8.3	0.84
Parenteral Feeding	(mL/kg/day)			
M2		84.8 ± 5.6	82.4 ± 5.6	0.92
M3		19.3 ± 9.0	12.2 ± 4.2	0.15
M4		7.5 ± 5.2	6.3 ± 4.2	0.54

Values are means ± standard error of the mean. CRIB (Clinical Risk Index for Babies); * Prenatal amniotic infection risk (Chorioamnionitis, maternal colonization of group streptococci (GBS), premature rupture of membranes, maternal fever in delivery). M2: 3rd day of postnatal life, M3: 15th day of postnatal life, M4: 30th day postnatal of life.

**Table 2:** Clinical outcomes at the time of discharge.

Clinical outcomes	Mother’s Milk Group	Control Group	*p*-Value
Days to achieve full enteral feeding *	7.2 ± 0.6	9.1 ± 0.7	0.04
Volume of full enteral feeding (mL) *	118.9 ± 5.3	107.8 ± 5.7	0.38
NEC at the end of the study (Bell stage ≥ 2)	2 (4.9%)	2 (4.3%)	1
Proven Sepsis at the end of the study	3 (7.3%)	2 (4.4%)	0.66
MV during 1st month of life	9 (21.9%)	13 (28.2%)	0.72
Abnormalities in ultrasound brain scan at 1st month of life	14 (34.1%)	10(21.7%)	0.11

* Data expressed as means ± standard error of the mean. NEC: necrotizing enterecolitis, MV: mechanical ventilation.
